# Supplementary material for: Synthetic lethality between VPS4A and VPS4B triggers an inflammatory response in colorectal cancer
Source: EMBO Mol Med. 2020 Jan 13;12(2):e10812. doi: 10.15252/emmm.201910812 (PMC7005644; doi:10.15252/emmm.201910812)
Supplement: Supplementary file 1 — Appendix [file EMMM-12-e10812-s001.pdf]

Synthetic lethality between VPS4A and VPS4B triggers an inflammatory response in colorectal cancer

Ewelina Szymańska et al.

APPENDIX FIGURES AND TABLES

Table of contents:

- **Appendix Figure S1.** Transcriptional alterations after individual or combined knockdown of VPS4 paralogs in HCT116 cells.
- **Appendix Figure S2**. Analyses of caspase activation in dying HCT116 cells upon chemical treatment or RelA depletion.
- **Appendix Figure S3**. Flow cytometry analysis of calreticulin cell surface exposure in VPS4A-depleted HCT116 *VPS4B*$^{-/-}$ cells.
- **Appendix Table S1**. List of selected terms after gene set enrichment analysis under conditions of combined silencing of *VPS4A+B*.
- **Appendix Table S2**. List of qRT-PCR primers used in this study.
- **Appendix Table S3**. Exact p-values for data from EV figures 1- 4.

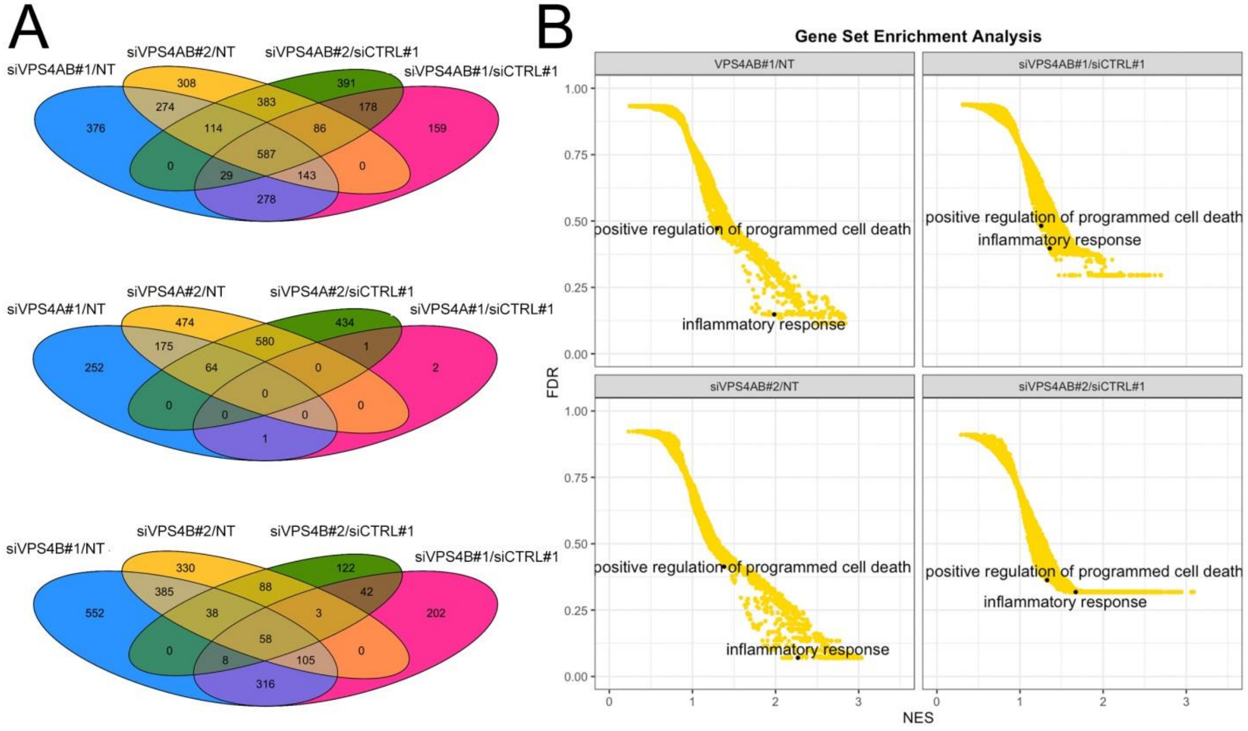

Appendix Figure S1. Transcriptional alterations after individual or combined knockdown of VPS4 paralogs in HCT116 cells.

A Venn diagrams of transcriptionally upregulated genes (≥ 1.5-fold; adjusted $p < 0.05$) after individual or combined *VPS4A* and/or *VPS4B* knockdown when normalized to non-transfected (NT) cells and cells transfected with non-targeting siRNA (siCTRL#1), as indicated. Transcriptionally upregulated genes were identified using DESeq2.

B Rank order based on Normalized Enrichment Score (NES) and False Discovery Rate (FDR) of enriched gene signatures for four different contrasts of combined *VPS4A+B* silencing and normalization conditions was prepared using the gseGO function from clusterProfiler.

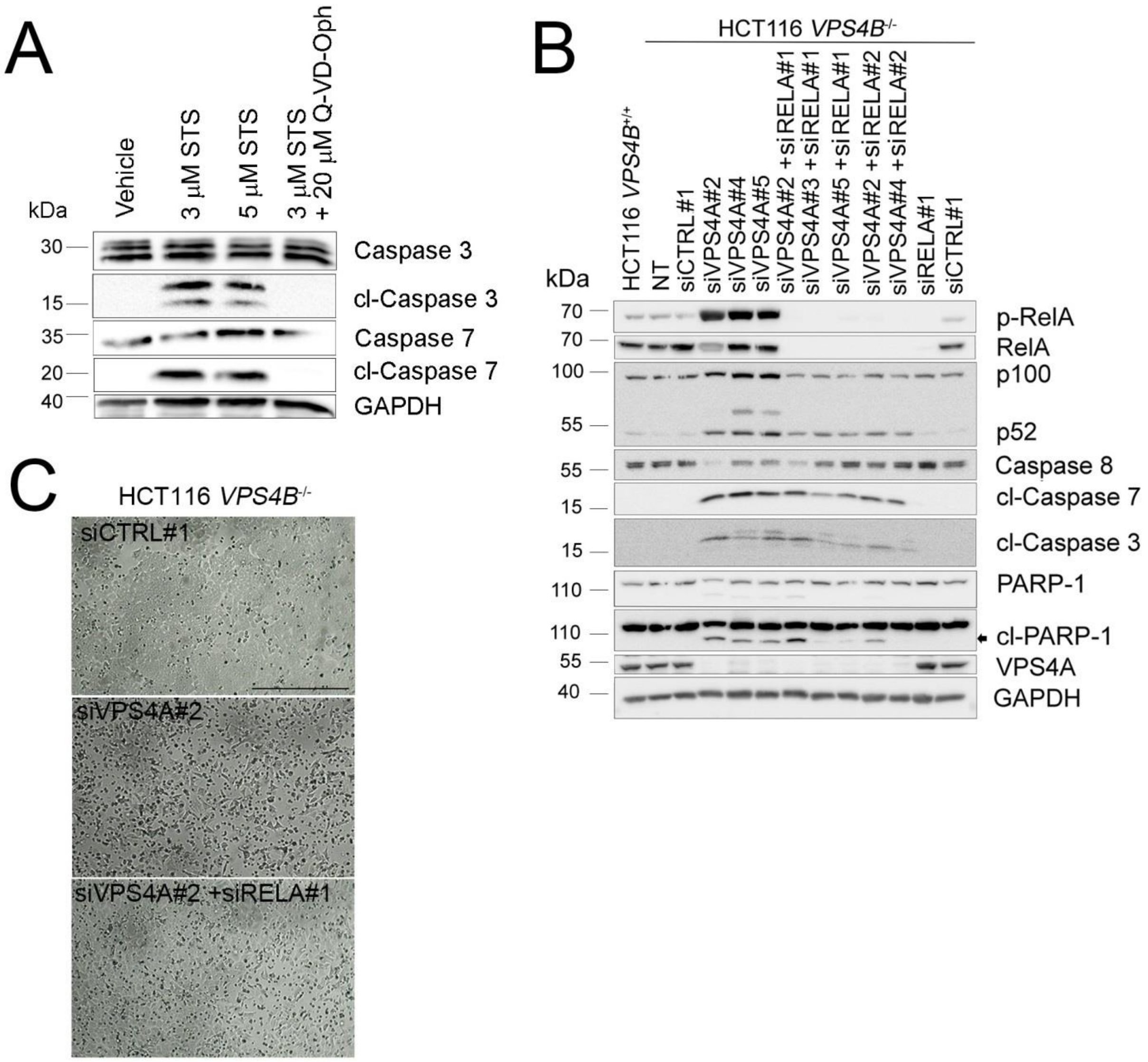

Appendix Figure S2. Analyses of caspase activation in dying HCT116 cells upon chemical treatment or RelA depletion.

A Immunoblotting analysis of caspase activation in apoptotic HCT116 cells**.** To induce apoptosis cells were treated for 24 h with staurosporine (STS). To prevent caspase activation, cells were pretreated with Q-VD-Oph 30 min before STS administration and then cultured for 24 h in the presence of STS and Q-VD-Oph. Vehicle – lysates from DMSO-treated cells. cl - cleaved caspases. GAPDH served as a loading control.

B Immunoblotting analysis of caspase activation in lysates from cells with depletion of both *VPS4* paralogs (as in Fig 5A) with or without co-depletion of RelA. Two independent siRNA oligonucleotides targeting *RELA* (siRELA#1 or #2) alone or in combination with siVPS4A were used for transfections. Representative blot from 3 experiments is shown. GAPDH served as a loading control. NT- non-transfected; p-RelA - phospho-RelA; cl – cleaved caspases or PARP-1.

C Phase contrast microscopy images of HCT116 *VPS4B*$^{-/-}$ cells transfected as in (B) acquired 66 h after siRNA transfection. Scale bar, 500 µm.

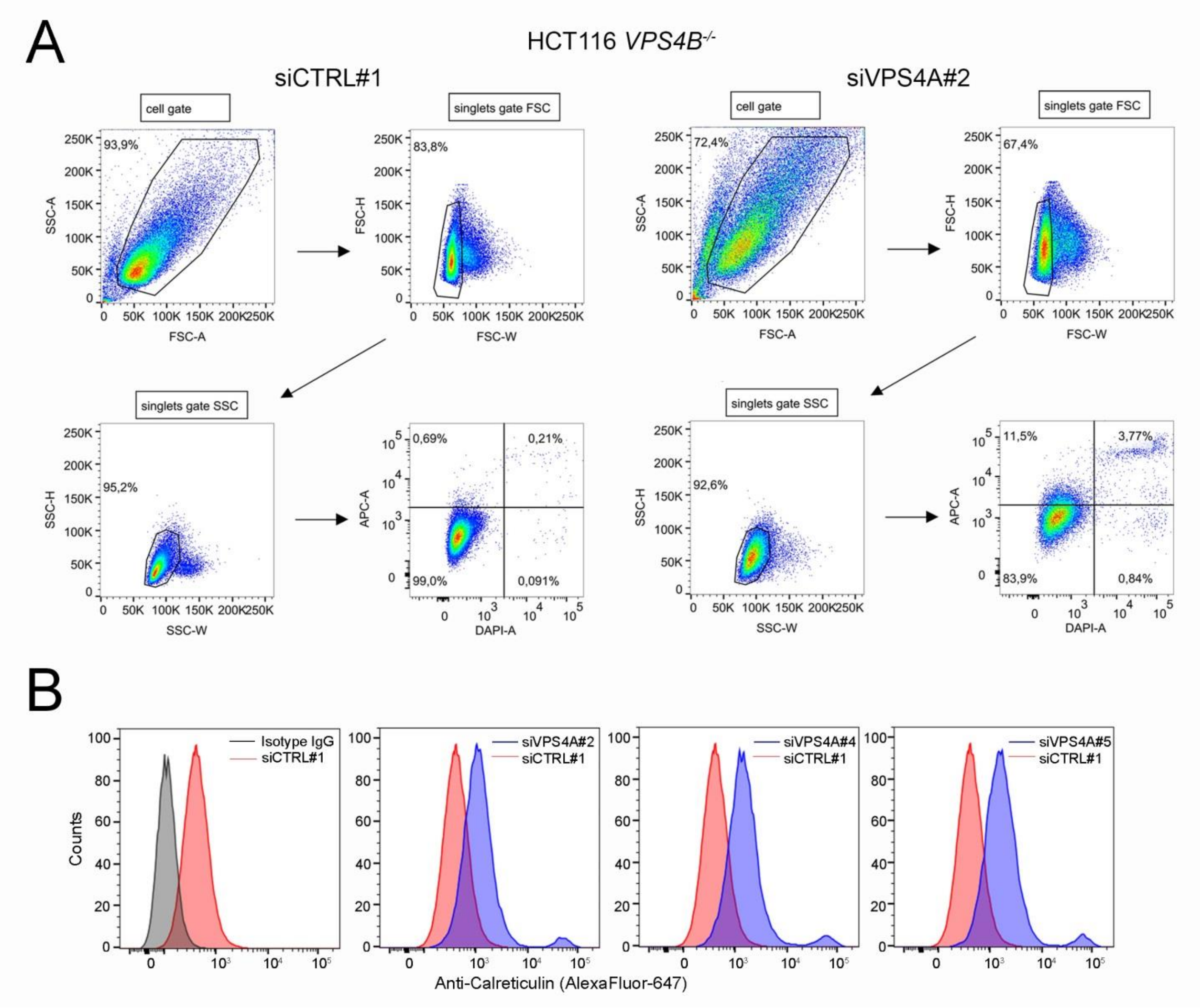

Appendix Figure S3. Flow cytometry analysis of calreticulin cell surface exposure in VPS4A-depleted HCT116 *VPS4B*$^{-/-}$ cells.

A Examples of gating strategy for flow cytometric analysis (Fig 6D), applied for siCTRL#1- (left) or siVPS4A#2-transfected (right) HCT116 *VPS4B*$^{-/-}$ cells, including cell gate (FSC vs SSC), singlets discrimination gates (FSC-H vs FSC-W, SSC-H vs SSC-W). DAPI staining was used to discriminate live (DAPI-) and dead cells (DAPI+). APC - AlexaFluor-647 fluorescence. This strategy was applied for all flow cytometry experiments.

B Overlay histograms showing fluorescence signal of AlexaFluor 647 indicating cell surface calreticulin exposure. One representative experiment from Fig 6D is shown. Grey histogram represents siCTRL#1-transfected cells stained with primary isotype IgG, followed by AlexaFluor 647-conjugated secondary IgG. Red histograms represent siCTRL#1-transfected cells and blue histograms represent cells transfected with siVPS4A (#2, #4, #5) which were stained with anti-calreticulin antibody, followed by AlexaFluor 647-conjugated secondary IgG.

ID	Description	NES	FDR	Type of contrast
GO:0006954	inflammatory response	1.356	0.387	VPS4AB#1/Ctrl
GO:0043068	positive regulation of programmed cell death	1.255	0.425	VPS4AB#1/Ctrl
GO:0006954	inflammatory response	1.669	0.320	VPS4AB#2/Ctrl
GO:0043068	positive regulation of programmed cell death	1.319	0.395	VPS4AB#2/Ctrl
GO:0006954	inflammatory response	1.984	0.148	VPS4AB#1/NT
GO:0043068	positive regulation of programmed cell death	1.297	0.472	VPS4AB#1/NT
GO:0006954	inflammatory response	2.271	0.100	VPS4AB#2/NT
GO:0043068	positive regulation of programmed cell death	1.389	0.408	VPS4AB#2/NT

Appendix Table S1. List of selected terms after gene set enrichment analysis under conditions of combined silencing of *VPS4A+B*.

Gene name	Forward primer	Reverse primer
ACTB	CAGGTCATCACCATTGGCAAT	TCTTTGCGGATGTCCACGT
B2M	GGAGGCTATCCAGCGTACTC	GAAACCCAGACACATAGCAATTC
VPS4A	CCATCAGGAGGAGGTTTGAA	CCGAGTAGCCTTCCGTCTTC
VPS4B	TCGTTAAATATGAAGCACAGGGTGA	TTCTCCTTCCCCATCACTGT
VPS4B-2	GAACTCCGCCATGTCATCC	TCGTAGTTCCCAGCCTTGTC
Nos2	TCAACTGCAAGAGAACGGAGA	TCTTTCAGGTCACTTTGGTAGGA
Cxcl9	TCGGACTTCACTCCAACACAG	AGGGTTCCTCGAACTCCACA
Arg1	CGTAGACCCTGGGGAACACTAT	TCCATCACCTTGCCAATCCC
Ym1	AGAAGCTCTCCAGAAGCAATCC	ATCAGCTGGTAGGAAGATCCCAG
Il10	GACTTTAAGGGTTACTTGGGTTGC	ATTTCTGGGCCATGCTTCTCT
Ccl22	AGGACTACATCCGTCACCCT	GACGGTTATCAAAACAACGCCA
Il18	CGACTTCACTGTACAACCGCA	TGGGGTTCACTGGCACTTTG
Il6	GATGGATGCTACCAAACTGG	TCTGAAGGACTCTGGCTTTG
Rpl19	AGGCATATGGGCATAGGGAAGAG	TTGACCTTCAGGTACAGGCTGTG
Vps4a	CAATTGATGGGTGCTGTTGTGA	GGCCCAAAGAGGAGTATGCC
Vps4b	AAATGCAAGGAGTTGGTGTGG	TCTAAACATGGCTGCTCGGG

Appendix Table S2. List of qRT-PCR primers used in this study.

Figures	Groups	Symbol	p-value	n	statistic test	post-test
**EV1A**	normal colon vs. adenoma	ns	0.4259	24 vs. 42	Kruskal-Wallis	Dunn's test with Benjamini-Hochberg correction
	normal colon vs. adenocarcinoma	ns	0.0628	24 vs. 26	Kruskal-Wallis	Dunn's test with Benjamini-Hochberg correction
	adenoma vs. adenocarcinoma	ns	0.1071	42 vs. 26	Kruskal-Wallis	Dunn's test with Benjamini-Hochberg correction
**EV2A**	VPS4A - siCTRL (#1 + #2) group vs. siVPS4B (#1 + #2) group	ns	0.5176	4	two-tailed unpaired t-test	
	VPS4B - siCTRL (#1 + #2) group vs. siVPS4A (#1 + #2) group	ns	0.3525	4	two-tailed unpaired t-test	
**EV2B**	VPS4A protein abundance - normalized siCTRL#1 (set as 1) vs. siVPS4B#1	ns	0.8750	4	Wilcoxon signed rank test	
	VPS4A protein abundance - normalized siCTRL#1 (set as 1) vs. siVPS4B#2	ns	0.6250	4	Wilcoxon signed rank test	
	VPS4B protein abundance - normalized siCTRL#1 (set as 1) vs. siVPS4A#1	ns	0.2500	4	Wilcoxon signed rank test	
	VPS4B protein abundance - normalized siCTRL#1 (set as 1) vs. siVPS4A#2	ns	0.6250	4	Wilcoxon signed rank test	
	VPS4B protein abundance - normalized siCTRL#1 (set as 1) vs. siVPS4A#3	ns	0.8750	4	Wilcoxon signed rank test	
**EV3B**	HCT116 *VPS4B* $^{+/+}$ vs. HCT116 *VPS4B* $^{-/-}$ 1C5	ns	0.6831	5	one-sample t-test	
	HCT116 *VPS4B* $^{+/+}$ vs. HCT 116 *VPS4B* $^{-/-}$ 2B3	ns	0.0681	5	one-sample t-test	
**EV3D**	siCTRL#1 vs. siVPS4A (#2, #4 and #5) group	****	8.79E-07	4	one-sample t-test	
**EV3F**	HCT116 VPS4B-/- Dox- vs. HCT116 VPS4B-/- Dox+	ns	0.4158	3	one-sample t-test	
	HCT116 VPS4B-/- shCTRL#1 Dox- vs. HCT116 VPS4B-/- shCTRL#1Dox+	ns	0.5672	3	two-tailed unpaired t-test	
	HCT116 VPS4B-/- shCTRL#2 Dox- vs. HCT116 VPS4B-/-shCTRL#2 Dox+	ns	0.4244	3	two-tailed unpaired t-test	
	HCT116 VPS4B-/- shVPS4A#1 Dox- vs. HCT116 VPS4B-/- shVPS4A#2 Dox+	**	0.0097	3	two-tailed unpaired t-test	
	HCT116 VPS4B-/- shVPS4A#2 Dox- vs. HCT116 VPS4B-/- skhVPS4A#2 Dox+	*	0.0208	3	two-tailed unpaired t-test	
**EV4A**	EEA1 NT vs. siVPS4A + VPS4B	**	0.0058		Welch t-test	
	Rab7 NT vs. siVPS4A + VPS4B	**	0.0100		Welch t-test	
	LAMP-1NT vs. siVPS4A + VPS4B	*	0.0430		the Mann-Whitney U test	
**EV4B**	(chart: % of Tf-A647 -positive cells) siCTRL#1 vs. siVPS4A#2 and #5 group	**	0.0034	4	two-tailed unpaired t-test	
	(chart: intensity of Tf-A647 -positive cells) siCTRL#1 vs. siVPS4A#2 and #5 group	***	0.0009	4	two-tailed unpaired t-test	
**EV4C**	G0/G1 siCTRL#1 vs. siVPS4A#2+siVPS4A5	**	0.0061	4	the Man -Whitney U test	
	S siCTRL#1 vs. siVPS4A#2+siVPS4A5	ns	0.1091	4	the Man -Whitney U test	
	G2/M siCTRL#1 vs .siVPS4A#2+siVPS4A5	**	0.0061	4	the Man -Whitney U test	

Appendix Table S3. Exact p-values for data from EV figures 1 - 4.
