# Supplementary material for: Synthetic lethality between VPS4A and VPS4B triggers an inflammatory response in colorectal cancer
Source: EMBO Mol Med. 2020 Jan 13;12(2):e10812. doi: 10.15252/emmm.201910812 (PMC7005644; doi:10.15252/emmm.201910812)
Supplement: Supplementary file 2 — Expanded View Figures PDF [file EMMM-12-e10812-s002.pdf]

# Expanded View Figures

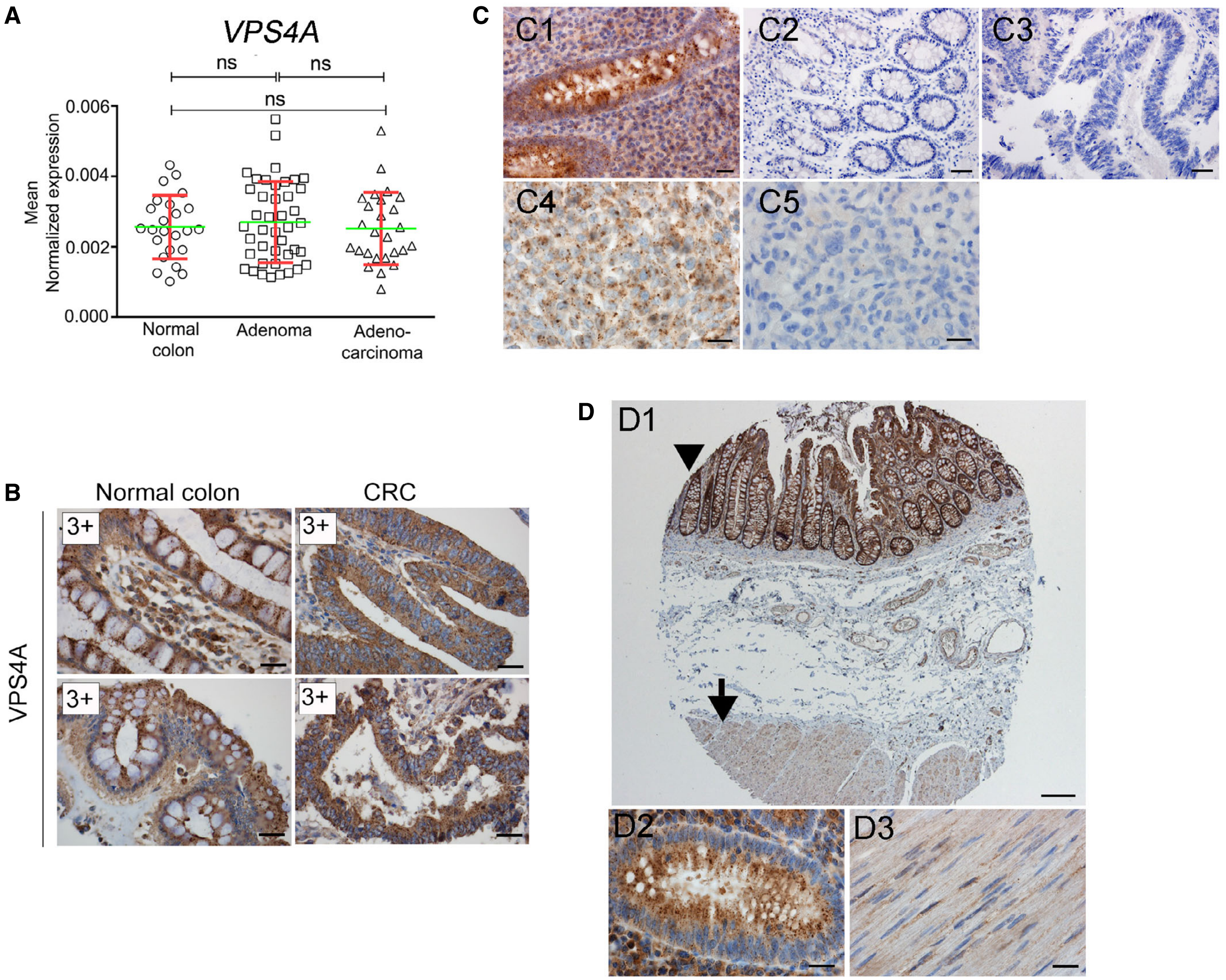


**Figure EV1. Evaluation of VPS4A and VPS4B protein abundance in human tissues and in CRC.**

A qPCR analysis of *VPS4A* mRNA abundance in normal colon, adenoma, and CRC samples. Adenocarcinoma ($n = 26$); adenoma ($n = 42$); normal colon ($n = 24$). Green horizontal bars indicate means, and red whiskers indicate SD. Differences were analyzed using the Kruskal–Wallis test followed by Dunn's multiple comparison *post hoc* test; ns—non-significant ($P \geq 0.05$).

B Immunohistochemistry (IHC) evaluation of VPS4A abundance in pairs of normal colon versus matched CRC samples (2 representative pairs out of 100 analyzed). 3+—very intensive staining. Scale bar, 20 μm.

C Specificity tests of VPS4B IHC staining using human tissues and mouse xenografts. C1—strong granular cytoplasmic staining in the mucosa of the appendix. C2, C3—negative staining in the appendix (C2) and CRC (C3) when primary antibody (anti-VPS4B) was omitted. C4—granular cytoplasmic staining in the xenograft from wild-type (*VPS4B*$^{+/+}$) HCT116 cells. C5—negative staining in the xenograft from HCT116 *VPS4B*$^{-/-}$ cells. Scale bars: C1, C4, and C5—20 μm; C2, C3—50 μm.

D Specificity tests of VPS4A IHC staining in various human tissues with high and low expression of *VPS4A*. D1—strong granular cytoplasmic staining in the mucosa of the appendix (arrowhead, and at a higher magnification in D2) and weak staining in the muscle (arrow, and at a higher magnification in D3). Scale bars: D1—200 μm; D2 and D3—20 μm.

Data information: The exact *P*-values can be found in Appendix Table S3.

 © 2020 The Authors

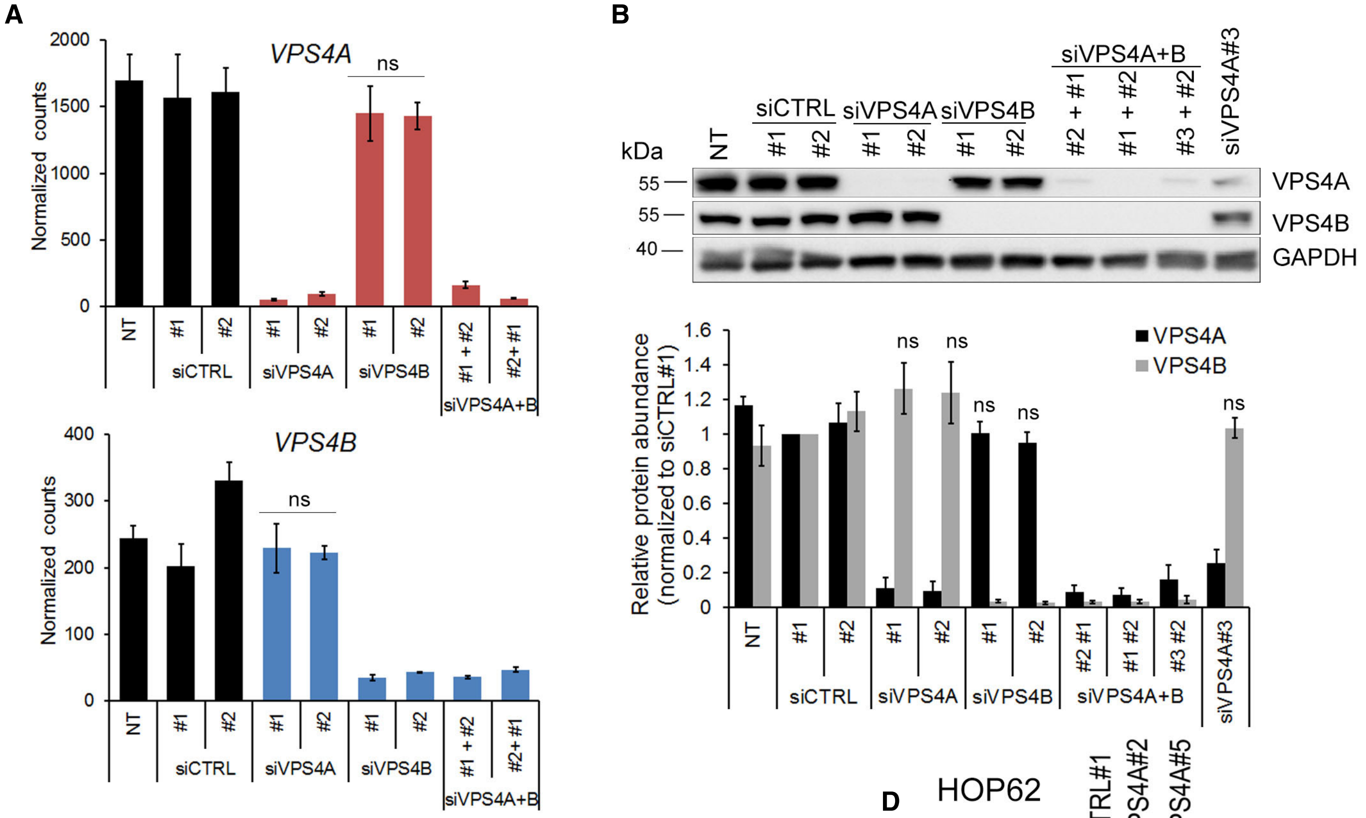


**C**

| Cell line | Cancer type | CRISPR (Avana) Public 19Q3 | | CRISPR (Sanger) | | Combined RNAi (Broad, Novartis, Marcotte) | | Copy number log2 (relative to ploidy +1) | |
|---|---|---|---|---|---|---|---|---|---|
| | | CERES | | | | DEMETER2 | | | |
| | | *VPS4A* | *VPS4B* | *VPS4A* | *VPS4B* | *VPS4A* | *VPS4B* | *VPS4B* | *VPS4A* |
| **SNU410** | **pancreas** | -1.67 | 0.1 | | | -1.53 | -0.12 | 0.48 * | 0.85 |
| **HOP62** | **lung** | -1.1 | 0.06 | -1.16 | 0.13 | -0.5 | -0.09 | 0.56 | 1.17 |
| **HCT116** | **colorectal** | | | -0.21 | -0.02 | 0.003 | -0.28 | 0.98 | 0.98 |
| **DLD1** | **colorectal** | -0.25 | -0.05 | | | 0.1 | -0.11 | 0.99 | 0.99 |
| **RKO** | **colorectal** | -0.23 | 0.06 | -0.26 | -0.002 | -0.12 | -0.04 | 0.96 | 1.03 |
| **A549** | **lung** | -0.3 | -0.03 | -0.22 | 0.05 | -0.02 | -0.35 | 0.84 | 1.03 |

* frameshift damaging insertion in *VPS4B* (p.A83fs)

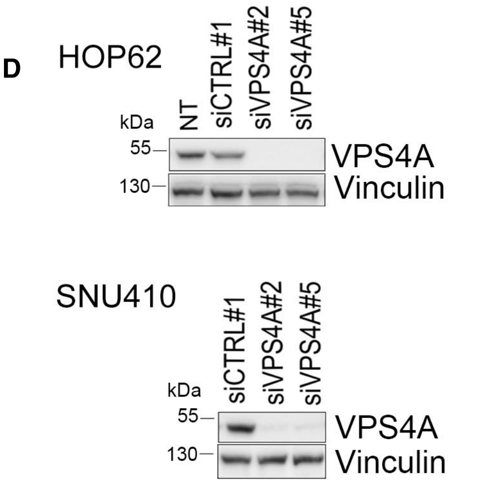


**Figure EV2. Analyses of *VPS4A* and *VPS4B* mRNA and protein abundance in HCT116 cells and the dependencies between both genes in different cell lines.**

A mRNA levels of *VPS4A* (upper panel) and *VPS4B* (lower panel) in HCT116 cells 72 h upon siRNA transfection. For VPS4A or VPS4B depletion, two different duplexes (#1 or #2) of siVPS4A or siVPS4B were used. For simultaneous VPS4A+B depletion, various combinations of siVPS4A and siVPS4B duplexes were used. Two different duplexes of siCTRL (#1 or #2) were used as non-targeting controls. NT—non-transfected. Values represent normalized counts after including variance normalized transformation performed by the DESeq2 package for RNA-Seq data analysis. Data are means of four independent experiments ± SEM. Two-tailed unpaired *t*-test; ns—non-significant ($P \geq 0.05$).

B Upper panel, representative immunoblotting analysis of VPS4A and VPS4B abundance in lysates of HCT116 cells collected 72 h after transfection as in (A). GAPDH was used as a loading control. Lower panel, densitometry analysis of VPS4A and VPS4B abundance based on immunoblotting analysis as shown in the upper panel. NT—non-transfected. Data are means of four independent experiments ± SEM. Wilcoxon signed rank test; ns—non-significant ($P \geq 0.05$).

C Correlation between dependency scores and *VPS4B* copy number for selected cancer cell lines from the DepMap portal dataset (https://depmap.org/portal/). According to the portal, a lower score (below −0.5) means that a gene is more likely to be dependent in a given cell line. A score of 0 is equivalent to a gene that is not essential, whereas a score of −1 corresponds to the median of all common essential genes.

D Immunoblotting analysis of *VPS4A* silencing efficiency in HOP62 and SNU410 cell lines. Lysates were prepared 6 days after siRNA transfection with non-targeting (siCTRL#1) or *VPS4A*-targeting (siVPS4A#2 or #5) duplexes. Vinculin was used as a loading control.

Data information: The exact *P*-values can be found in Appendix Table S3.

© 2020 The Authors

**Figure EV3. Characterization of HCT116 *VPS4B*$^{-/-}$ cell line and engineering of HCT116 VPS4B$^{-/-}$ cells with doxycycline (Dox)-inducible *VPS4A*-targeting shRNA expression (HCT116 *VPS4B*$^{-/-}$ shVPS4A) for *in vivo* studies.**

A Left panel, PCR sequencing analysis verifying bi-allelic *VPS4B* knockout in two CRISPR/Cas9 engineered clones derived from the HCT116 *VPS4B*$^{+/+}$ parental line. Right panel, immunoblotting analysis of VPS4B in cell lysates of these clones.

B Comparison of clonal growth of isogenic HCT116 lines: parental *VPS4B*$^{+/+}$ and *VPS4B*$^{-/-}$. Upper panel, analysis of colony area of parental HCT116 *VPS4B*$^{+/+}$ cells and *VPS4B*$^{-/-}$ clones assessed in the colony formation assay. Data are means of five independent experiments. Values were normalized to the colony area of parental HCT116 *VPS4B*$^{+/+}$ cells that was set as 1. Error bars are SEM. One-sample *t*-test; ns—non-significant ($P \geq 0.05$). Lower panel, representative images of clonal growth of parental and *VPS4B*$^{-/-}$ HCT116 cells that were used for the quantification presented on the top.

C Analysis of the tumor growth in mice bearing parental HCT116 *VPS4B*$^{+/+}$ or *VPS4B*$^{-/-}$ xenografts. $n = 3$ mice for each group, each mouse bearing two tumors, ± SEM.

D Confirmation of cell death of HCT116 *VPS4B*$^{-/-}$ cells upon VPS4A depletion. Upper panel, viability of HCT116 *VPS4B*$^{-/-}$ assessed 72 h after siVPS4A transfection (three independent siVPS4A duplexes #2, #4, and #5 were used). Non-transfected (NT) or siCTRL#1-transfected cells served as viability controls. Data are means of four independent experiments. Values were normalized to the viability of siCTRL#1-transfected cells that was set as 100%. Error bars are SEM. One-sample *t*-test; ****$P < 0.0001$. Lower panel, representative images of HCT116 *VPS4B*$^{-/-}$ clones grown for 15 days after siCTRL#1 or siVPS4A transfection. NT—non-transfected cells.

E Immunoblotting analysis of VPS4A abundance in lysates of HCT116 *VPS4B*$^{-/-}$ shVPS4A clones (#1 to #3, each bearing an independent *VPS4A*-targeting shRNA construct). To induce shRNA expression, doxycycline (Dox) was administered to the cell culture medium (1 μg/ml) 3 days before cell lysis. Lysates of doxycycline-treated and non-treated HCT116 *VPS4B*$^{-/-}$ shCTRL clones (#1 and #2, each bearing an independent non-targeting shRNA construct) were loaded as controls. Lysates from HCT116 *VPS4B*$^{+/+}$ cells were used to control VPS4A and VPS4B protein detection. GAPDH served as a loading control.

F Comparison of the growth rate of HCT116 *VPS4B*$^{-/-}$ line and its derivative clones bearing different shVPS4A (#1 and #2) or shCTRL (#1 and #2) constructs cultured in the presence (+Dox) or absence (−Dox) of doxycycline. Data are expressed as the percentage of the viability of HCT116 *VPS4B*$^{-/-}$ cultured in the absence of doxycycline at day 5 (set as 100%). Values are means of three independent experiments. Error bars are SEM. Two-tailed unpaired *t*-test; ns—non-significant ($P \geq 0.05$), *$P < 0.05$; **$P < 0.01$.

G Monitoring of body weight of mice bearing engrafted HCT116 *VPS4B*$^{-/-}$ shVPS4A#1 cells. Day 1 indicates the first day of doxycycline administration (+Dox) to the drinking water of mice. Data represent the means of body weight of 9 mice used in each experimental condition. Error bars are SEM.

Data information: The exact *P*-values can be found in Appendix Table S3.


© 2020 The Authors

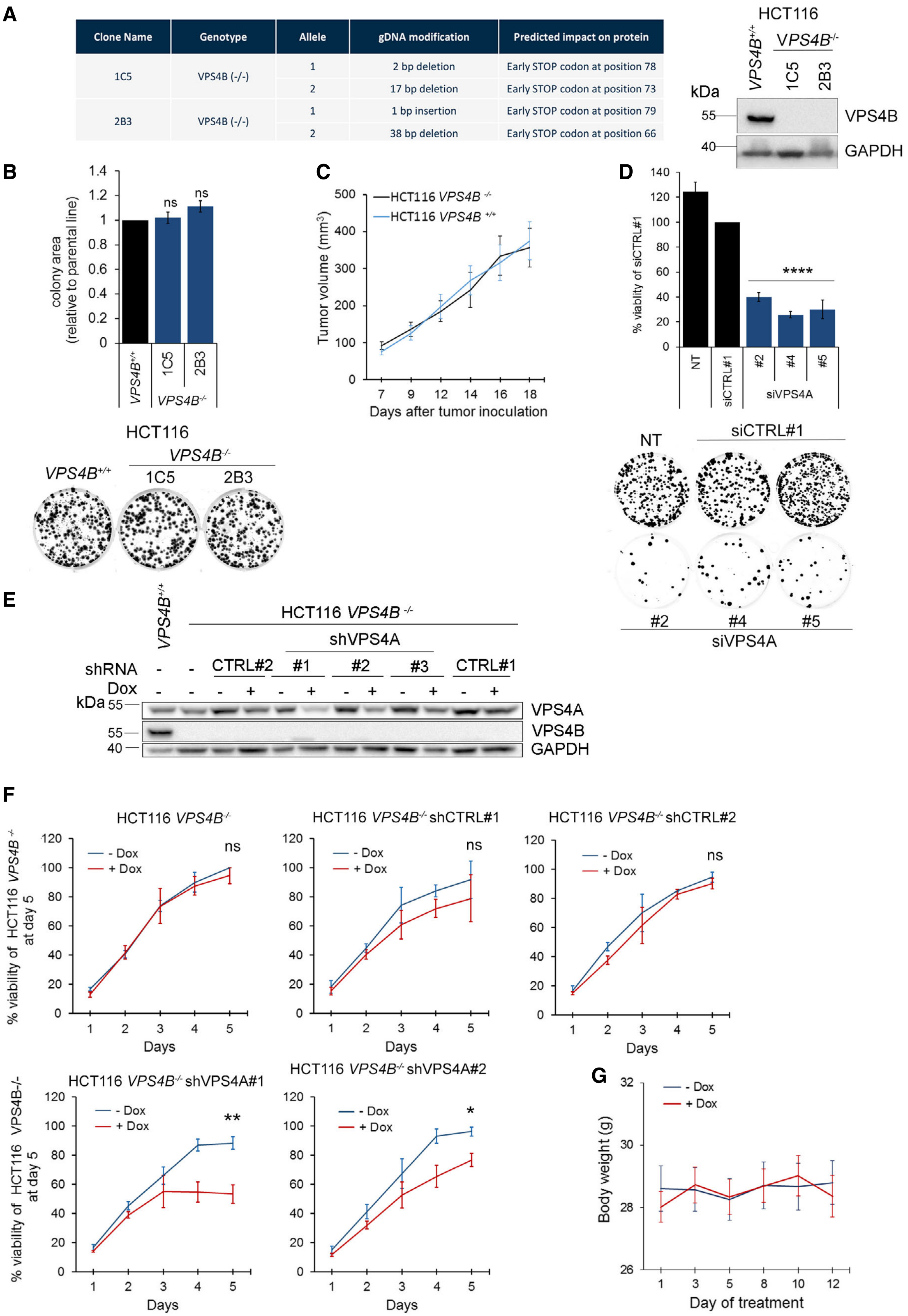


| Clone Name | Genotype | Allele | gDNA modification | Predicted impact on protein |
|---|---|---|---|---|
| 1C5 | VPS4B (-/-) | 1 | 2 bp deletion | Early STOP codon at position 78 |
| | | 2 | 17 bp deletion | Early STOP codon at position 73 |
| 2B3 | VPS4B (-/-) | 1 | 1 bp insertion | Early STOP codon at position 79 |
| | | 2 | 38 bp deletion | Early STOP codon at position 66 |

**Figure EV3.**

**Figure EV4. Inhibition of endocytosis and cell cycle progression upon simultaneous depletion of VPS4A and VPS4B.**

A Upper panel, microscopy images of HCT116 cells collected 48 h after transfection with control (siCTRL#1) or *VPS4A*- and/or *VPS4B*-targeting siRNA (siVPS4A#2 or #5 and siVPS4B#1 or #2). EEA1, Rab7, and LAMP1 were used as markers of early endosomes (EE), late endosomes (LE), and lysosomes, respectively, and were visualized in green. Nuclei were stained with Hoechst 33342 (blue). NT—non-transfected cells. Scale bar, 10 μm. Lower panel, quantified fluorescence signals from microscopy images. The boxes denote the $25^{th}$ to $75^{th}$ percentile range, the center lines mark the $50^{th}$ percentile (median) and the whiskers reflect the largest and smallest observed values in at least four z-stacks from three independent experiments. The Welch *t*-test (for EEA1 and Rab7) and the Mann–Whitney *U*-test (for LAMP1); *$P < 0.05$; **$P < 0.01$.

B Upper panel, example of flow cytometry analysis of AlexaFluor 647-labeled transferrin (Tf-A647) uptake by non-transfected (NT) or siRNA (siCTRL#1 and siVPS4A#2 or #5) transfected HCT116 $VPS4B^{-/-}$ cells. Lower panel presents the percentage and mean fluorescence intensity of Tf-A647-positive cells. Data are means of four independent experiments ± SEM. Fluorescence intensity of non-transfected Tf-A647-treated cells was set as 1. Statistical significance was assessed using the Mann–Whitney *U*-test and *t*-test. **$P < 0.01$; ***$P < 0.001$.

C Left panel, example of flow cytometry analysis of cell cycle phase distribution in HCT116 $VPS4B^{-/-}$ cells 72 h after transfection with control (siCTRL#1) or *VPS4A*-targeting siRNA (siVPS4A#2 and #5). NT—non-transfected. Graphs were generated using the ModFit program. Right panel, analysis of cell cycle phase distribution in HCT116 $VPS4B^{-/-}$ cells transfected with siRNA as indicated. Data are means of four independent experiments ± SEM. Statistical significance was assessed using the Mann–Whitney *U*-test. ns—non-significant ($P \geq 0.05$); **$P < 0.01$.

Data information: The exact *P*-values can be found in Appendix Table S3.

 © 2020 The Authors

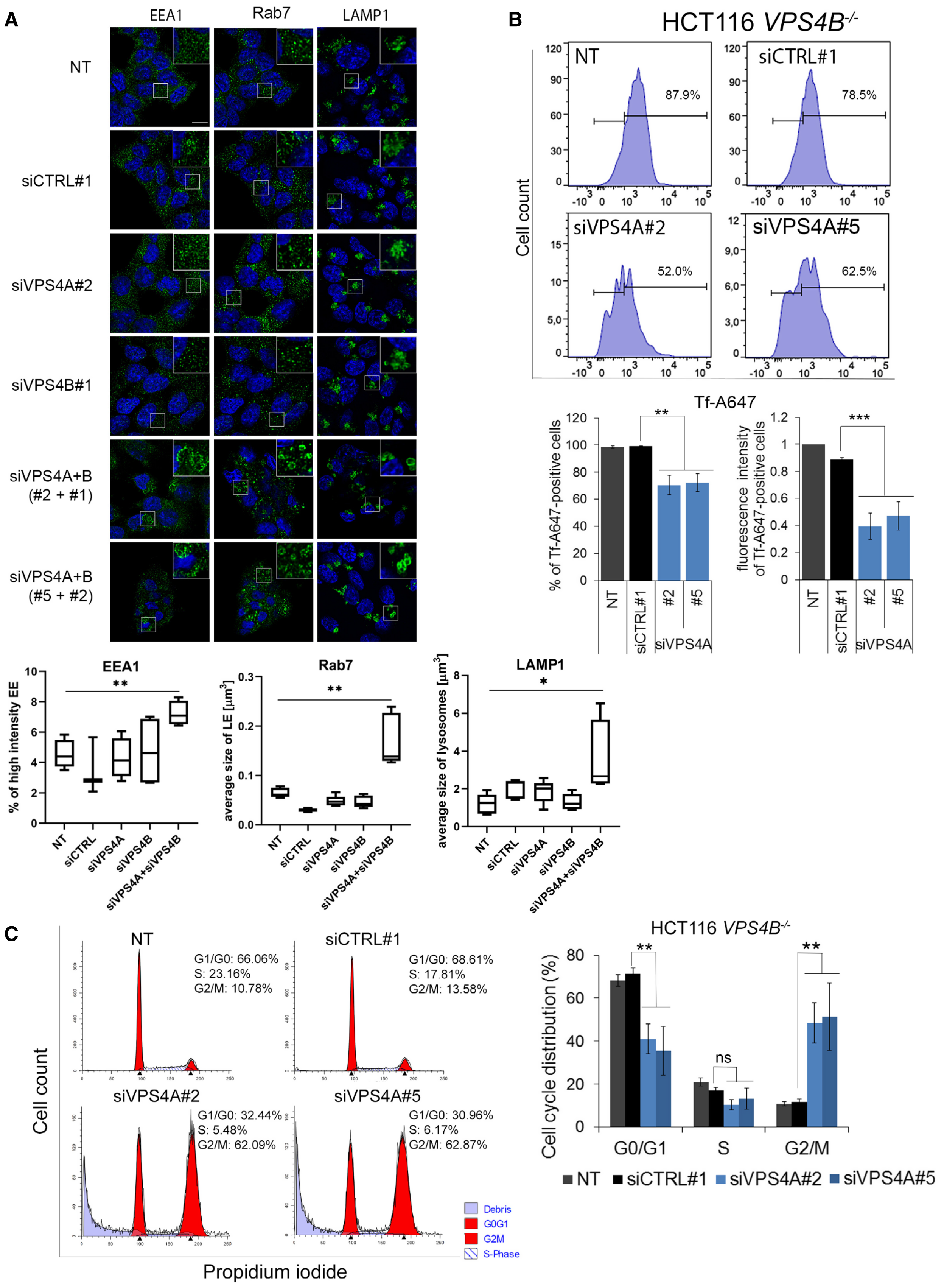
A
EEA1
Rab7
LAMP1
NT
siCTRL#1
siVPS4A#2
siVPS4B#1
siVPS4A+B (#2 + #1)
siVPS4A+B (#5 + #2)
B
HCT116 VPS4B-/-
Cell count
87.9%
78.5%
52.0%
62.5%
Tf-A647
% of Tf-A647-positive cells
fluorescence intensity of Tf-A647-positive cells
siVPS4A
EEA1
% of high intensity EE
Rab7
average size of LE [µm³]
LAMP1
average size of lysosomes [µm³]
NT
siCTRL
siVPS4A
siVPS4B
siVPS4A+siVPS4B
C
G1/G0: 66.06%
S: 23.16%
G2/M: 10.78%
G1/G0: 68.61%
S: 17.81%
G2/M: 13.58%
G1/G0: 32.44%
S: 5.48%
G2/M: 62.09%
G1/G0: 30.96%
S: 6.17%
G2/M: 62.87%
Debris
G0G1
G2M
S-Phase
Propidium iodide
Cell cycle distribution (%)
G0/G1
S
G2/M
ns
NT siCTRL#1 siVPS4A#2 siVPS4A#5

**Figure EV4.**

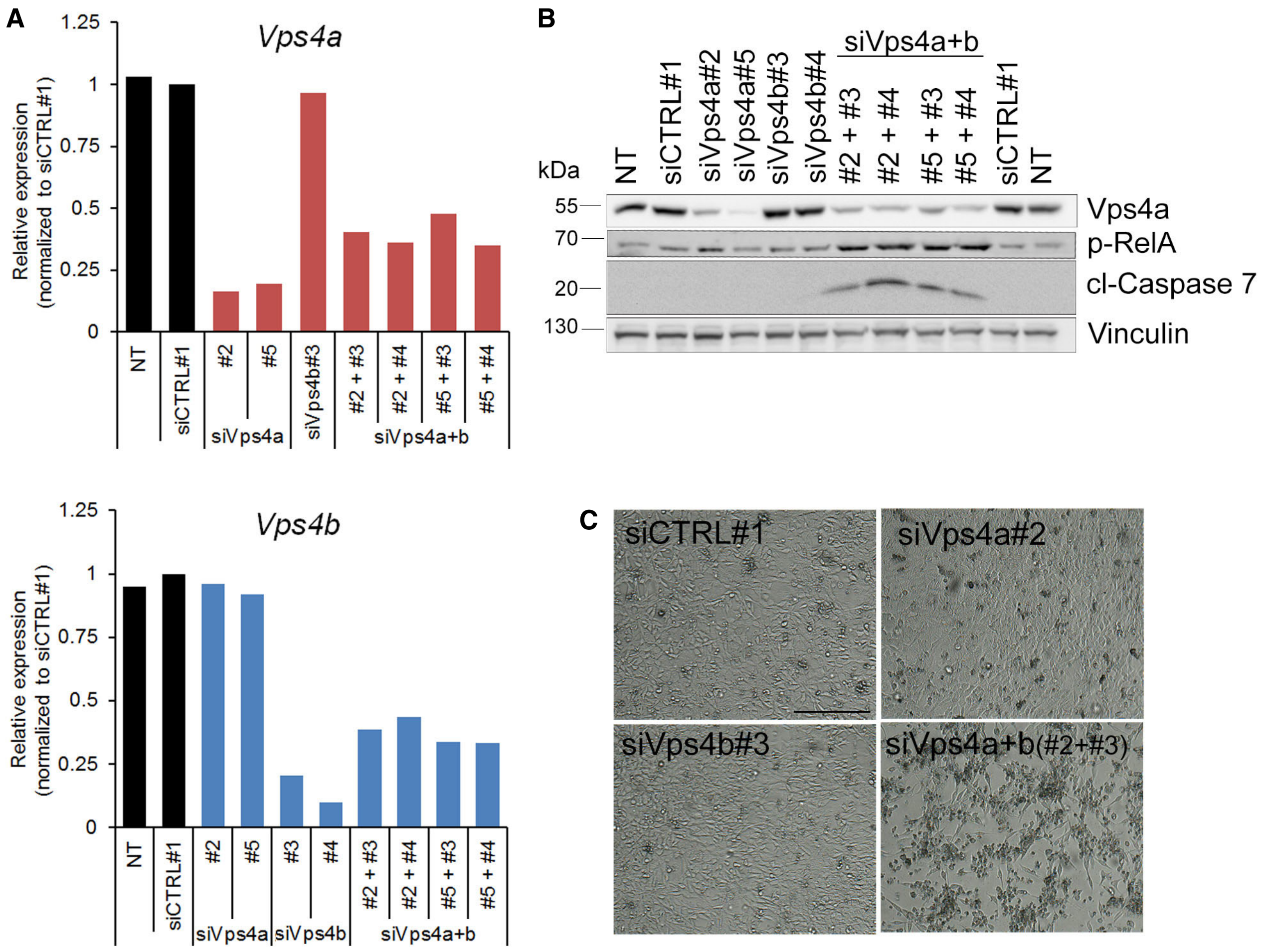


**Figure EV5. (supporting Fig 6). Combined depletion of Vps4a+b in mouse colon carcinoma CT-26 cells.**

A qRT–PCR analysis of the silencing efficiency of *Vps4a* (upper panel) and *Vps4b* (lower panel) in CT-26 cells 72 h after transfection with siRNA. To deplete Vps4a or Vps4b, two independent siRNA duplexes were used (#2 or #5, and #3 or #4, respectively). To simultaneously deplete Vps4a+b, various combinations of siVps4a and siVps4b were used. All values were normalized; *Vps4a* or *Vps4b* expression values in siCTRL#1-transfected cells were set as 1 and used to normalize mRNA abundance in other conditions. NT—non-transfected.

B Immunoblotting detection of Vps4a abundance, inflammatory response (phosphorylated RelA) and apoptosis activation (cleaved caspase 7) in lysates of mouse CT-26 cells collected 72 h after siRNA transfection as in (A). p-RelA—phosphorylated RelA; cl—cleaved caspase. Vinculin served as a loading control.

C Phase contrast microscopy images of CT-26 cells acquired 3 days after transfection with siVps4a or siVps4b as described in (A). Scale bar, 250 μm.


© 2020 The Authors
