# Supplementary material for: Synthetic lethality between VPS4A and VPS4B triggers an inflammatory response in colorectal cancer
Source: EMBO Mol Med. 2020 Jan 13;12(2):e10812. doi: 10.15252/emmm.201910812 (PMC7005644; doi:10.15252/emmm.201910812)
Supplement: Supplementary file 8 — Source Data for Figure 2 [file EMMM-12-e10812-s007.zip › Szymanska_et_al_Legends_for_Source_data_Fig_2_.docx]

**Synthetic lethality between VPS4A and VPS4B triggers**

**an inflammatory response in colorectal cancer**

**Ewelina Szymańska et al.**

**Source data for Fig. 2**

**Table of contents:**

1. File **“Szymanska et al Fig 2 statistics”** contains exact p-values for data in Fig. 2
2. File  **“Szymanska et al Fig 2F WB source data”** contains uncropped immunoblot images from Fig. 2F
