# Supplementary figures and images for: Synthetic lethality between VPS4A and VPS4B triggers an inflammatory response in colorectal cancer
Source: EMBO Mol Med. 2020 Jan 13;12(2):e10812. doi: 10.15252/emmm.201910812 (PMC7005644; doi:10.15252/emmm.201910812)

Figure 2F

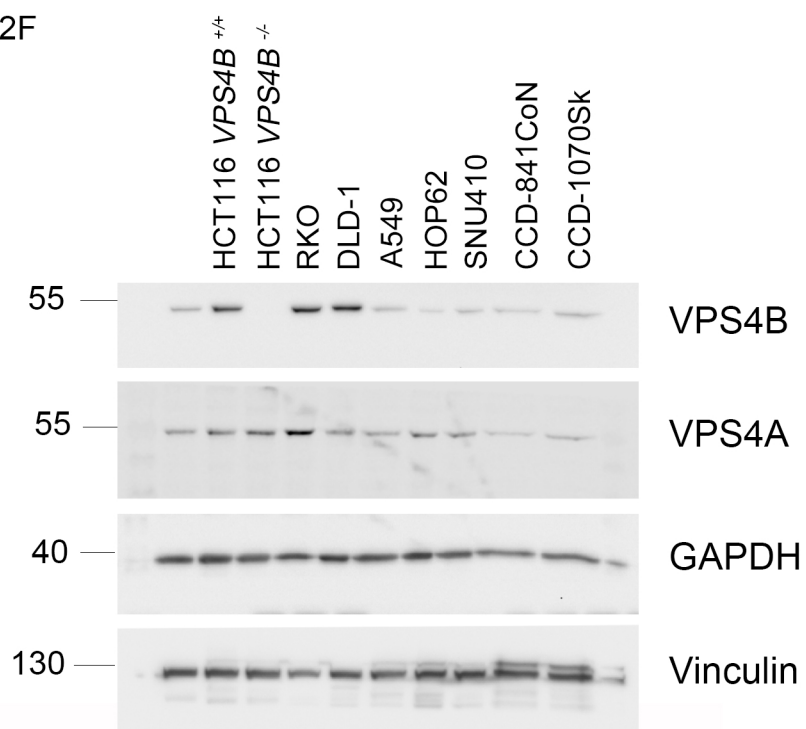

Supplement: Supplementary file 8 — Source Data for Figure 2 [file EMMM-12-e10812-s007.zip › Szymanska_et_al__Fig_2F_WB_source_data_.pdf]

Figure 3C

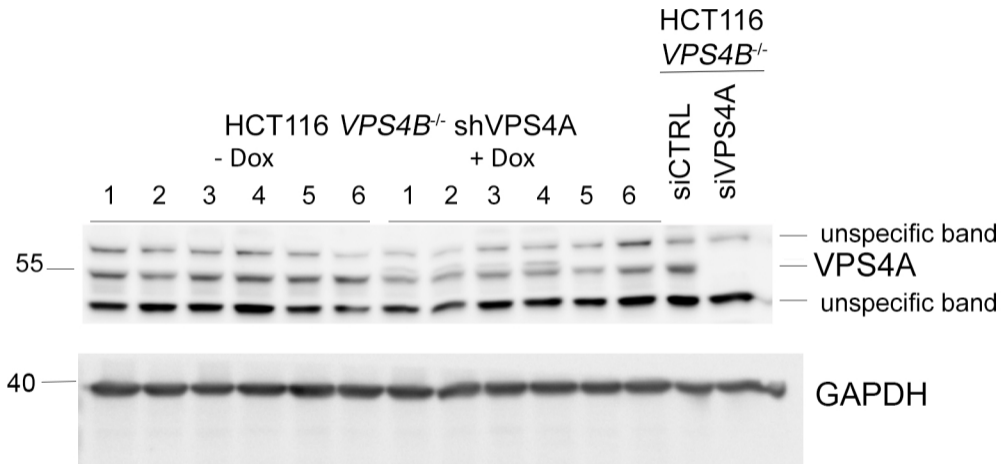

Supplement: Supplementary file 9 — Source Data for Figure 3 [file EMMM-12-e10812-s008.zip › Szymanska_et_al_Fig_3C_WB_source_data_.pdf]

Figure 5A

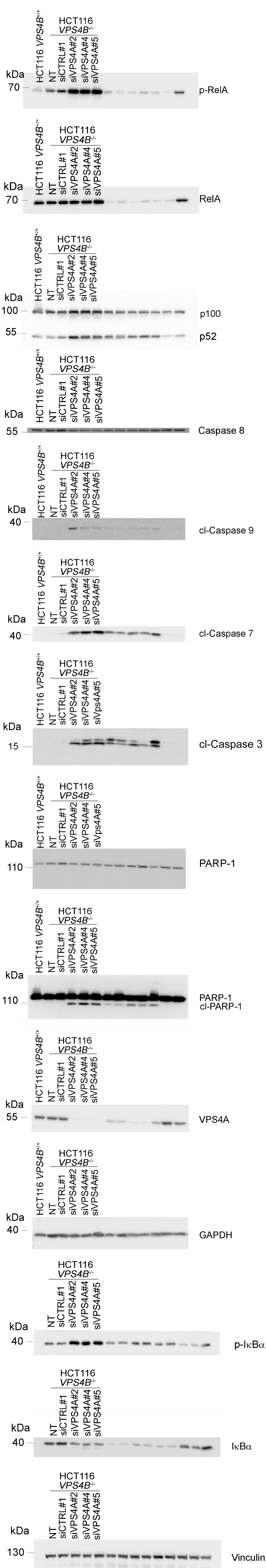

Supplement: Supplementary file 10 — Source Data for Figure 5 [file EMMM-12-e10812-s009.zip › Szymanska_et_al_Fig_5A_WB_source_data.pdf]

Figure 5C

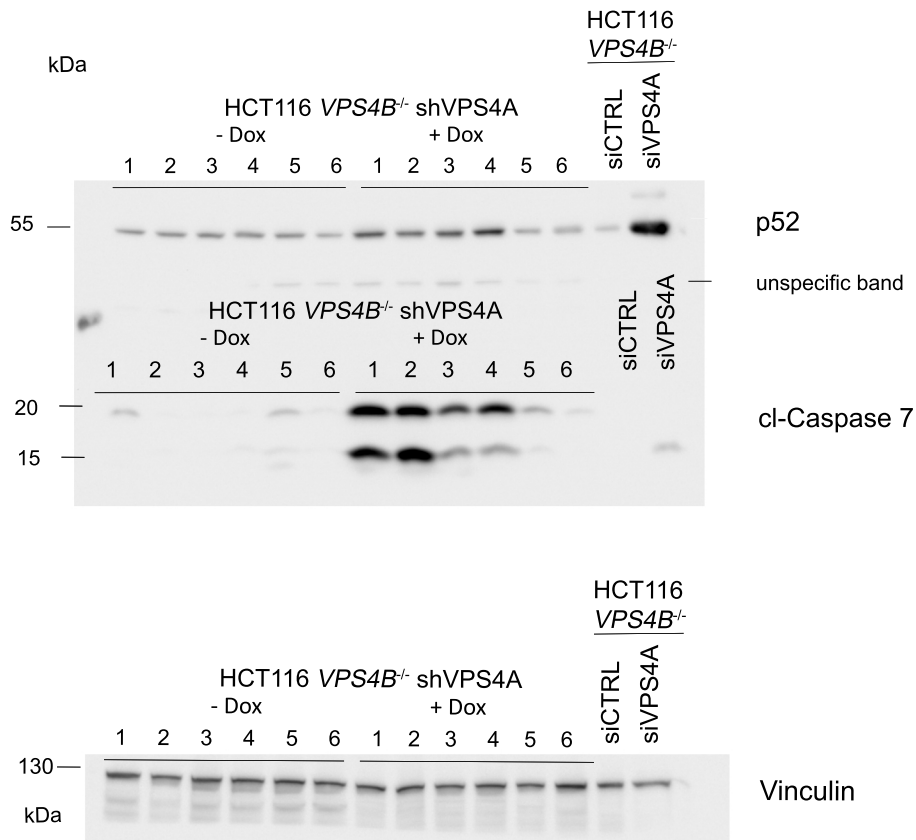

Supplement: Supplementary file 10 — Source Data for Figure 5 [file EMMM-12-e10812-s009.zip › Szymanska_et_al_Fig_5C_WB_source_data.pdf]
